# Supplementary material for: Does working from home work? That depends on the home
Source: PLoS One. 2024 Aug 7;19(8):e0306475. doi: 10.1371/journal.pone.0306475 (PMC11305525; doi:10.1371/journal.pone.0306475)
Supplement: S1 Table — (DOCX) [file pone.0306475.s001.docx]

|  | WFH-HWQ Productivity | Self-Reported Productivity | WFH-HWQ Stress & Irritability Factor |
| --- | --- | --- | --- |
| Self-Reported Productivity  (Single-item Scale) | .73 |  |  |
| WFH-HWQ Stress & Irritability Factor | -.55 | -.37 |  |
| Burnout Tendency (MBI Scale) | -.50 | -.31 | .71 |
