# Supplementary material for: Does working from home work? That depends on the home
Source: PLoS One. 2024 Aug 7;19(8):e0306475. doi: 10.1371/journal.pone.0306475 (PMC11305525; doi:10.1371/journal.pone.0306475)
Supplement: S2 Table — (DOCX) [file pone.0306475.s002.docx]

|  | Desk  Home | Chair  Home | Screen  Home | Hardware Home | Wi-Fi  Home | Temperature Home | Air Quality Home | Lighting Home | Noise  Home |
| --- | --- | --- | --- | --- | --- | --- | --- | --- | --- |
| **Panel 1: Home Satisfaction** | | | | | | | | | |
| Chair Home | .70 |  |  |  |  |  |  |  |  |
| Screen Home | .57 | .58 |  |  |  |  |  |  |  |
| Hardware Home | .50 | .52 | .72 |  |  |  |  |  |  |
| Wi-Fi Home | .36 | .39 | .46 | .55 |  |  |  |  |  |
| Temperature Home | .36 | .33 | .36 | .39 | .43 |  |  |  |  |
| Air Quality Home | .35 | .38 | .39 | .45 | .42 | .60 |  |  |  |
| Lighting Home | .36 | .32 | .35 | .37 | .37 | .53 | .60 |  |  |
| Noise Home | .35 | .36 | .37 | .40 | .37 | .41 | .48 | .41 |  |
| **Panel 2: Work Satisfaction** | | | | | | | | | |
| Desk Work | .09 | .21 | .18 | .24 | .21 | .14 | .23 | .14 | .21 |
| Chair Work | .12 | .17 | .19 | .25 | .22 | .19 | .25 | .18 | .20 |
| Screen Work | .07 | .17 | .17 | .25 | .27 | .19 | .27 | .19 | .22 |
| Hardware Work | .14 | .22 | .26 | .37 | .32 | .21 | .29 | .19 | .26 |
| Wi-Fi Work | .17 | .24 | .23 | .33 | .28 | .18 | .24 | .18 | .25 |
| Temperature Work | .10 | .14 | .11 | .15 | .13 | .06 | .14 | .12 | .05 |
| Air Quality Work | .13 | .15 | .15 | .17 | .13 | .13 | .17 | .13 | .08 |
| Lighting Work | .10 | .17 | .20 | .19 | .15 | .19 | .24 | .17 | .16 |
| Noise Work | .06 | .08 | .12 | .12 | .14 | .15 | .18 | .13 | .04 |
