# Supplementary material for: Does working from home work? That depends on the home
Source: PLoS One. 2024 Aug 7;19(8):e0306475. doi: 10.1371/journal.pone.0306475 (PMC11305525; doi:10.1371/journal.pone.0306475)
Supplement: S3 Table — (DOCX) [file pone.0306475.s003.docx]

|  | Dependent variable: Productivity | | | | |
| --- | --- | --- | --- | --- | --- |
|  | (1) | (2) | (3) | (4) | (5) |
| Desk & Chair | .15 (.04)*** |  | .09 (.04)** | .09 (.04)** | .12 (.05)** |
| Screen & Hardware | .18 (.05)*** |  | .11 (.05)** | .11 (.05)** | .12 (.05)** |
| WiFi | .18 (.03)*** |  | .10 (.04)*** | .10 (.04)*** | .07 (.04) |
| Temperature |  | .14 (.04)*** | .09 (.05)** | .10 (.05)** | .10 (.06)** |
| Air Quality |  | .08 (.04)* | .03 (.04) | .04 (.04) | .08 (.05) |
| Lighting |  | .11 (.04)*** | .07 (.04)* | .06 (.04) | .04 (.05) |
| Noise |  | .21 (.04)*** | .16 (.04)*** | .16 (.04)*** | .13 (.04)*** |
| Age (years) | .01 (.003)*** | .01 (.003)*** | .01 (.003)*** | .01 (.003)*** | .01 (.003)*** |
| Income (Baseline: Modal) |  |  |  |  |  |
| Mininum wage (less than 11,000) | -.68 (.24)*** | -.56 (.24)*** | -.58 (.23)*** | -.58 (.24)*** | -.46 (.28)** |
| below modal (11-23k) | -.10 (.10) | -.03 (.10) | -.04 (.10) | -.06 (.10) | -.05 (.11) |
| 1-2x modal (34-56k) | -.12 (.08) | -.10 (.08) | -.10 (.08) | -.09 (.08) | -.08 (.09) |
| 2x modal or more (56k) | -.14 (.09) | -.11 (.09) | -.12 (.09) | -.12 (.09) | -.02 (.10) |
| don’t know/ don’t want to say | .01 (.09) | .04 (.09) | .01 (.09) | -.01 (.09) | -.01 (.10) |
| Female | .21 (.07)*** | .10 (.07) | .15 (.07)** | .14 (.07)** | .15 (.08)** |
| Household Members | .10 (.04)** | .12 (.04)*** | .11 (.04)*** | .09 (.04)** | .07 (.05) |
| Children Home during Office Hours (baseline: no children) | |  |  |  |  |
| Always | -.70 (.19)*** | -.65 (.18)*** | -.63 (.18)*** | -.63 (.18)*** | -.74 (.21)*** |
| Sometimes | -.22 (.10)** | -.23 (.10)** | -.22 (.09)** | -.17 (.09)* | -.17 (.10) |
| Never | -.07 (.10) | -.07 (.10) | -.07 (.10) | -.06 (.10) | -.07 (.11) |
| Partner Home during Office Hours (baseline: no Partner) | |  |  |  |  |
| Always | .06 (.09) | .06 (.09) | .06 (.09) | .06 (.09) | .02 (.10) |
| Sometimes | .17 (.09)* | .11 (.09) | .15 (.08)* | .13 (.09) | .13 (.10) |
| Never | .20 (.09)** | .11 (.09) | .14 (.08)* | .15 (.09)* | .10 (.10) |
| Pet (Baseline: No pets) |  |  |  |  |  |
| Dog | .10 (.07) | .09 (.06) | .07 (.06) | .07 (.07) | .08 (.08) |
| Cat | -.02 (.06) | -.01 (.07) | -.02 (.06) | -.04 (.06) | -.01 (.08) |
| Company size (Baseline: 0-5) |  |  |  |  |  |
| 5-15 | .01 (.17) | -.04 (.16) | -.05 (.16) | -.09 (.17) | -.16 (.20) |
| 15-50 | .10 (.15) | .02 (.14) | .02 (.14) | -.02 (.15) | -.12 (.18) |
| 50+ | -.003 (.14) | -.03 (.14) | -.04 (.14) | -.08 (.14) | -.14 (.18) |
| Work Sector (Baseline: Governmental) |  |  |  |  |  |
| Yes, non-governmental | -.03 (.07) | .02 (.07) | .0005 (.07) | -.01 (.07) | -.01 (.08) |
| Yes, temp/ on-call worker | .03 (.17) | .14 (.17) | .09 (.16) | .09 (.17) | .05 (.17) |
| Yes, self-employed | -.04 (.15) | .01 (.14) | -.03 (.14) | -.10 (.15) | -.14 (.17) |
| Contract hours (Baseline: Full time (36+) |  |  |  |  |  |
| 20-35 hours | -.02 (.07) | -.03 (.07) | -.03 (.07) | -.01 (.07) | .004 (.08) |
| 12-19 hours | .20 (.13) | .16 (.13) | .18 (.13) | .20 (.13) | .19 (.14) |
| less than 12 hours | -.05 (.13) | -.18 (.12) | -.12 (.12) | -.11 (.13) | -.01 (.13) |
| Work suitable to perform from home | .05 (.03) | .07 (.03)** | .04 (.03) | .04 (.03) | .06 (.04)* |
| Home Office Floor plan (Baseline: Average) | |  |  |  |  |
| Open |  |  |  | .02 (.10) | .05 (.11) |
| Closed |  |  |  | -.07 (.09) | -.02 (.11) |
| Home Office Lighting (Baseline: Average) | |  |  |  |  |
| Natural |  |  |  | .02 (.09) | .01 (.11) |
| No Natural |  |  |  | -.01 (.17) | -.11 (.21) |
| Home Office Ventilation (Baseline: None) | |  |  |  |  |
| Mechanic |  |  |  | -.07 (.16) | -.17 (.18) |
| Manual |  |  |  | -.08 (.14) | -.11 (.16) |
| Home Office surface (m^2^) |  |  |  | -.03 (.03) | -.05 (.03) |
| Real-estate value (x€1,000) |  |  |  |  | -.01 (.04) |
| Address-density (per kilometer radius) |  |  |  |  | -.06 (.07) |
| Urbanicity (Baseline: Extremely high) |  |  |  |  |  |
| High |  |  |  |  | -.10 (.13) |
| Moderate |  |  |  |  | -.06 (.16) |
| Low |  |  |  |  | .0003 (.18) |
| None-Urban |  |  |  |  | -.04 (.21) |
| Observations | 1,002 | 1,002 | 1,002 | 956 | 734 |
| R2 | .25 | .27 | .30 | .30 | .30 |
| Adjusted R2 | .23 | .25 | .28 | .27 | .26 |
| Residual Std. Error | .88 (df = 972) | .87 (df = 971) | .85 (df = 968) | .85 (df = 915) | .83 (df = 687) |
| F Statistic | 11.41*** (df = 29; 972) | 12.16*** (df = 30; 971) | 12.79*** (df = 33; 968) | 10.01*** (df = 40; 915) | 6.50*** (df = 46; 687) |
| *Note.* **p*<0.1, ***p*<0.05, ****p*<0.01. | | | | | |
