# Supplementary material for: Does working from home work? That depends on the home
Source: PLoS One. 2024 Aug 7;19(8):e0306475. doi: 10.1371/journal.pone.0306475 (PMC11305525; doi:10.1371/journal.pone.0306475)
Supplement: S4 Table — (DOCX) [file pone.0306475.s004.docx]

|  | Dependent variable: Burnout Tendency | | | | | | | | |  |
| --- | --- | --- | --- | --- | --- | --- | --- | --- | --- | --- |
|  | (1) | (2) | | (3) | | (4) | | (5) | |  |
| Desk & Chair | -.14 (.04)*** |  | | -.10 (.04)** | | -.11 (.05)** | | -.16 (.06)*** | |  |
| Screen & Hardware | -.09 (.05)* |  | | -.04 (.05) | | -.03 (.05) | | -.001 (.06) | |  |
| WiFi | -.12 (.04)*** |  | | -.07 (.04)* | | -.07 (.04)* | | -.05 (.05) | |  |
| Temperature |  | -.07 (.04)* | | -.04 (.04) | | -.06 (.04) | | -.06 (.06) | |  |
| Air Quality |  | -.11 (.04)** | | -.08 (.04)* | | -.09 (.04)** | | -.07 (.05) | |  |
| Lighting |  | -.05 (.04) | | -.02 (.04) | | .01 (.04) | | .02 (.05) | |  |
| Noise |  | -.13 (.04)*** | | -.10 (.04)*** | | -.09 (.04)** | | -.09 (.05)** | |  |
| Age (years) | -.01 (.003)*** | -.01 (.003)*** | | -.01 (.003)*** | | -.01 (.003)** | | -.01 (.003)* | |  |
| Income (Baseline: Modal) |  |  | |  | |  | |  | |  |
| Mininum wage (less than 11,000) | .63 (.26)*** | .56 (.27)*** | | .56 (.27)*** | | .57 (.28)*** | | .46 (.32)* | |  |
| below modal (11-23k) | .26 (.14)** | .22 (.14)* | | .23 (.14)** | | .27 (.14)** | | .24 (.17)* | |  |
| 1-2x modal (34-56k) | -.01 (.08) | -.02 (.08) | | -.02 (.08) | | -.02 (.08) | | -.05 (.10) | |  |
| 2x modal or more (56k) | .13 (.10) | .12 (.10) | | .13 (.09) | | .12 (.10) | | .07 (.11) | |  |
| don’t know/ don’t want to say | .21 (.11)** | .20 (.11)* | | .21 (.11)** | | .23 (.11)** | | .24 (.13)** | |  |
| Female | .01 (.07) | .09 (.07) | | .05 (.07) | | .09 (.07) | | .03 (.08) | |  |
| Household Members | -.10 (.04)** | -.11 (.05)** | | -.10 (.04)** | | -.09 (.05)* | | -.08 (.05) | |  |
| Children Home during Office Hours (baseline: no children) | | |  | |  | |  | |  | |
| Always | .42 (.22)** | .42 (.22)** | | .39 (.22)** | | .35 (.21)* | | .42 (.24)* | |  |
| Sometimes | .06 (.10) | .07 (.10) | | .06 (.10) | | .02 (.10) | | -.02 (.11) | |  |
| Never | -.24 (.09)** | -.23 (.09)** | | -.23 (.09)** | | -.25 (.09)** | | -.26 (.11)** | |  |
| Partner Home during Office Hours (baseline: no Partner) | | |  | |  | |  | |  | |
| Always | .08 (.09) | .08 (.09) | | .08 (.09) | | .09 (.10) | | .13 (.11) | |  |
| Sometimes | -.07 (.10) | -.05 (.09) | | -.07 (.09) | | -.03 (.10) | | -.11 (.11) | |  |
| Never | -.14 (.09) | -.09 (.09) | | -.11 (.09) | | -.10 (.10) | | -.10 (.11) | |  |
| Pet (Baseline: No pets) |  |  | |  | |  | |  | |  |
| Dog | .15 (.08)** | .16 (.08)** | | .17 (.08)** | | .19 (.08)** | | .17 (.10)* | |  |
| Cat | -.002 (.07) | -.01 (.07) | | .001 (.07) | | .01 (.07) | | -.02 (.08) | |  |
| Company size (Baseline: 0-5) |  |  | |  | |  | |  | |  |
| 5-15 | -.03 (.15) | .02 (.14) | | .02 (.14) | | .02 (.15) | | .04 (.19) | |  |
| 15-50 | -.15 (.14) | -.10 (.13) | | -.10 (.13) | | -.06 (.14) | | -.10 (.18) | |  |
| 50+ | .16 (.14) | .18 (.13) | | .18 (.13) | | .20 (.14) | | .16 (.17) | |  |
| Work Sector (Baseline: Governmental) |  |  | |  | |  | |  | |  |
| Yes, non-governmental | .11 (.07) | .07 (.07) | | .08 (.07) | | .09 (.08) | | .15 (.08)* | |  |
| Yes, temp/ on-call worker | -.05 (.20) | -.15 (.19) | | -.11 (.19) | | -.12 (.22) | | -.10 (.25) | |  |
| Yes, self-employed | -.09 (.13) | -.14 (.13) | | -.10 (.13) | | -.05 (.14) | | -.09 (.16) | |  |
| Contract hours (Baseline: Full time (36+) | |  | |  | |  | |  | |  |
| 20-35 hours | .01 (.07) | .02 (.07) | | .02 (.07) | | -.02 (.07) | | .03 (.08) | |  |
| 12-19 hours | -.15 (.14) | -.14 (.13) | | -.14 (.14) | | -.16 (.14) | | -.03 (.15) | |  |
| less than 12 hours | -.33 (.14)** | -.25 (.15) | | -.29 (.15)* | | -.32 (.16)* | | -.49 (.17)** | |  |
| Work suitable to perform from home | -.01 (.03) | -.03 (.03) | | -.01 (.03) | | -.01 (.03) | | .01 (.04) | |  |
| Home Office Floor plan (Baseline: Average) | | |  | |  | |  | |  | |
| Open |  |  | |  | | .07 (.10) | | .04 (.11) | |  |
| Closed |  |  | |  | | .07 (.09) | | .04 (.11) | |  |
| Home Office Lighting (Baseline: Average) | |  | |  | |  | |  | |  |
| Natural |  |  | |  | | -.16 (.10) | | -.15 (.12) | |  |
| No Natural |  |  | |  | | .10 (.19) | | -.01 (.20) | |  |
| Home Office Ventilation (Baseline: None) | |  | |  | |  | |  | |  |
| Mechanic |  |  | |  | | .17 (.19) | | .14 (.22) | |  |
| Manual |  |  | |  | | .03 (.17) | | .01 (.19) | |  |
| Home Office surface (m^2^) |  |  | |  | | .03 (.03) | | .04 (.04) | |  |
| Real-estate value (x€1,000) |  |  | |  | |  | | .01 (.04) | |  |
| Address-density (per kilometer radius) |  |  | |  | |  | | -.03 (.06) | |  |
| Urbanicity (Baseline: Extremely high) |  |  | |  | |  | |  | |  |
| High |  |  | |  | |  | | -.02 (.13) | |  |
| Moderate |  |  | |  | |  | | -.02 (.16) | |  |
| Low |  |  | |  | |  | | -.15 (.16) | |  |
| None-Urban |  |  | |  | |  | | -.14 (.20) | |  |
| Observations | 1,002 | 1,002 | | 1,002 | | 956 | | 734 | |  |
| R2 | .18 | .19 | | .21 | | .21 | | .21 | |  |
| Adjusted R2 | .16 | .17 | | .18 | | .17 | | .16 | |  |
| Residual Std. Error | .92 (df = 972) | .91 (df = 971) | | .91 (df = 968) | | .90 (df = 915) | | .91 (df = 687) | |  |
| F Statistic | 7.60*** (df = 29; 972) | 7.71*** (df = 30; 971) | | 7.70*** (df = 33; 968) | | 6.06*** (df = 40; 915) | | 3.93*** (df = 46; 687) | |  |

*Note.* **p*<0.1, ***p*<0.05, ****p*<0.01.
