# Supplementary material for: Does working from home work? That depends on the home
Source: PLoS One. 2024 Aug 7;19(8):e0306475. doi: 10.1371/journal.pone.0306475 (PMC11305525; doi:10.1371/journal.pone.0306475)
Supplement: S5 Table — (DOCX) [file pone.0306475.s005.docx]

|  | Dependent variable: Willingness to continue working from home | | | | |
| --- | --- | --- | --- | --- | --- |
|  | (1) | (2) | (3) | (4) | (5) |
| Desk & Chair | .14 (.04)*** |  | .11 (.04)** | .11 (.05)** | .09 (.06)* |
| Screen & Hardware | .0004 (.05) |  | -.03 (.05) | -.02 (.05) | .05 (.06) |
| WiFi | .12 (.04)*** |  | .07 (.04)** | .08 (.04)** | .06 (.04) |
| Temperature |  | .12 (.04)*** | .09 (.04)** | .09 (.04)** | .07 (.05) |
| Air Quality |  | .02 (.05) | .004 (.05) | -.01 (.05) | .01 (.06) |
| Lighting |  | .08 (.04)** | .06 (.04)* | .08 (.04)* | .03 (.05) |
| Noise |  | .05 (.04) | .03 (.04) | .01 (.04) | -.002 (.05) |
| Age (years) | .01 (.003)*** | .01 (.003)*** | .01 (.003)*** | .01 (.003)*** | .01 (.003)** |
| Income (Baseline: Modal) |  |  |  |  |  |
| Mininum wage (less than 11,000) | -.17 (.19) | -.14 (.20) | -.13 (.19) | -.06 (.20) | -.01 (.24) |
| below modal (11-23k) | .03 (.11) | .08 (.11) | .07 (.11) | .08 (.11) | .11 (.12) |
| 1-2x modal (34-56k) | -.11 (.08) | -.10 (.08) | -.11 (.08) | -.10 (.08) | -.09 (.10) |
| 2x modal or more (56k) | -.16 (.09)* | -.14 (.09) | -.14 (.09) | -.15 (.10) | -.08 (.11) |
| don’t know/ don’t want to say | -.02 (.10) | -.005 (.10) | -.01 (.10) | -.02 (.10) | .01 (.12) |
| Female | .09 (.06) | .05 (.06) | .07 (.06) | .06 (.07) | .05 (.08) |
| Household Members | -.004 (.05) | .01 (.04) | .004 (.04) | .01 (.05) | -.01 (.05) |
| Children Home during Office Hours (baseline: no children) | |  |  |  |  |
| Always | -.23 (.19) | -.24 (.18) | -.21 (.19) | -.28 (.20) | -.32 (.23) |
| Sometimes | -.10 (.10) | -.12 (.09) | -.10 (.10) | -.13 (.10) | -.07 (.11) |
| Never | .03 (.09) | .03 (.09) | .03 (.09) | .03 (.09) | -.02 (.11) |
| Partner Home during Office Hours (baseline: no Partner) | |  |  |  |  |
| Always | .07 (.09) | .09 (.09) | .09 (.09) | .11 (.10) | .07 (.11) |
| Sometimes | .12 (.09) | .11 (.09) | .11 (.09) | .14 (.09) | .11 (.11) |
| Never | .14 (.09) | .11 (.09) | .12 (.09) | .15 (.09) | .08 (.11) |
| Pet (Baseline: No pets) |  |  |  |  |  |
| Dog | .09 (.07) | .09 (.07) | .08 (.07) | .07 (.07) | .03 (.09) |
| Cat | -.04 (.07) | -.04 (.06) | -.04 (.06) | -.04 (.07) | -.02 (.08) |
| Company size (Baseline: 0-5) | |  |  |  |  |
| 5-15 | -.25 (.15) | -.28 (.15)* | -.27 (.15)* | -.25 (.16) | -.15 (.18) |
| 15-50 | -.31 (.14)** | -.34 (.13)** | -.34 (.14)** | -.29 (.14)** | -.23 (.17) |
| 50+ | -.36 (.12)*** | -.37 (.12)*** | -.37 (.13)*** | -.34 (.13)** | -.25 (.16) |
| Work Sector (Baseline: Governmental) | |  |  |  |  |
| Yes, non-governmental | .05 (.07) | .08 (.07) | .08 (.07) | .08 (.07) | .07 (.09) |
| Yes, temp/ on-call worker | -.03 (.14) | .03 (.15) | -.01 (.14) | -.09 (.16) | -.12 (.21) |
| Yes, self-employed | .08 (.13) | .09 (.14) | .07 (.14) | .10 (.14) | .04 (.16) |
| Contract hours (Baseline: Full time (36+) | |  |  |  |  |
| 20-35 hours | .01 (.07) | .01 (.07) | .01 (.07) | .03 (.07) | .05 (.08) |
| 12-19 hours | -.05 (.14) | -.09 (.14) | -.08 (.14) | -.04 (.15) | .02 (.16) |
| less than 12 hours | -.01 (.14) | -.09 (.14) | -.06 (.14) | -.05 (.15) | -.24 (.18) |
| Work suitable to perform from home | .32 (.03)*** | .34 (.03)*** | .33 (.03)*** | .32 (.03)*** | .34 (.04)*** |
| Home Office Floor plan (Baseline: Average) | |  |  |  |  |
| Open |  |  |  | -.02 (.09) | -.03 (.10) |
| Closed |  |  |  | -.12 (.09) | -.16 (.10) |
| Home Office Lighting (Baseline: Average) | |  |  |  |  |
| Natural |  |  |  | .01 (.09) | .08 (.11) |
| No Natural |  |  |  | .21 (.18) | .21 (.21) |
| Home Office Ventilation (Baseline: None) | |  |  |  |  |
| Mechanic |  |  |  | .22 (.18) | .18 (.20) |
| Manual |  |  |  | .04 (.16) | .03 (.18) |
| Home Office surface (m^2^) |  |  |  | .01 (.03) | -.02 (.04) |
| Real-estate value (x€1,000) |  |  |  |  | -.01 (.03) |
| Address-density (per kilometer radius) |  |  |  |  | -.02 (.06) |
| Urbanicity (Baseline: Extremely high) | |  |  |  |  |
| High |  |  |  |  | .21 (.12)* |
| Moderate |  |  |  |  | .01 (.15) |
| Low |  |  |  |  | .02 (.17) |
| None-Urban |  |  |  |  | .05 (.19) |
| Observations | 1,002 | 1,002 | 1,002 | 956 | 734 |
| R2 | .24 | .25 | .26 | .26 | .26 |
| Adjusted R2 | .22 | .22 | .23 | .23 | .21 |
| Residual Std. Error | .88 (df = 972) | .88 (df = 971) | .88 (df = 968) | .88 (df = 915) | .88 (df = 687) |
| F Statistic | 10.65*** (df = 29; 972) | 10.63*** (df = 30; 971) | 10.13*** (df = 33; 968) | 8.03*** (df = 40; 915) | 5.24*** (df = 46; 687) |

*Note.* **p*<0.1, ***p*<0.05, ****p*<0.01.
