# Supplementary figures and images for: Does working from home work? That depends on the home
Source: PLoS One. 2024 Aug 7;19(8):e0306475. doi: 10.1371/journal.pone.0306475 (PMC11305525; doi:10.1371/journal.pone.0306475)

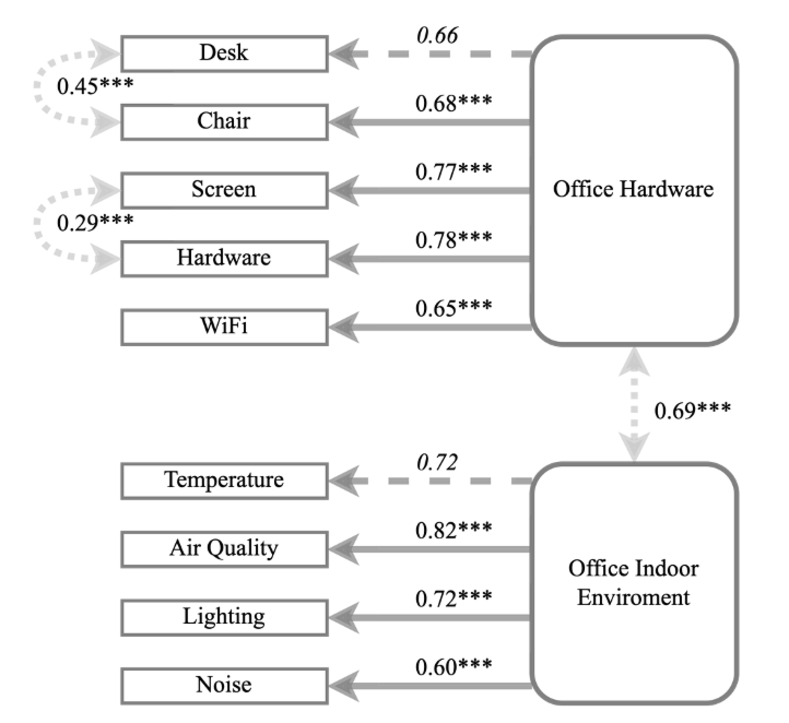

Supplement: S1 Fig — (TIF) [file pone.0306475.s006.tif]
