# Supplementary material for: Does working from home work? That depends on the home
Source: PLoS One. 2024 Aug 7;19(8):e0306475. doi: 10.1371/journal.pone.0306475 (PMC11305525; doi:10.1371/journal.pone.0306475)
Supplement: Attachment — Submitted filename: Response to Referees R&R3.pdf [file pone.0306475.s008.pdf]

Response to Referees

Reviewer #2

- Thank you for the revisions. The manuscript reads significantly better.

  We thank the Reviewer for the kind words

- Couple minor edits:

  Note that in Table 1., the value for high education level is missing.

  We have noted this mistake and amended the table.

- Line 208: It is unclear what is meant by manual of mechanical ventilation, and 0% to 100%. Is "manual" the ability to change the air flow or fan, or is it simply opening the window? Or do the authors actually mean air quality? Or perhaps the authors mean 'no' ventilation vs. 'having' manual or mechanical ventilation. Please clarify in the main text.

  We have added a note in-text to clarify the distinction between manual and mechanical ventilation. Specifically:

  "The former included layout of the home office (open versus closed), lighting (natural light versus no natural light), and ventilation ***(none, mechanical systems like HVAC or fans, or manual methods such as opening windows or doors)****. Additionally, participants were asked to estimate the surface of their home office (length and width in meters), and how often they ventilated their home office* ***(as a percentage of time spent in the home office).****"*

Note that, although this distinction is mainly important to check whether the participants had agency regarding their decision to ventilate, we do not treat mechanically or manually ventilated offices differently throughout the paper. We also do not require the participants to estimate the air quality, beyond their satisfaction level with the air quality. We have adjusted our conclusion regarding the effect of more ventilation to a broader concept:

"We find that higher home office satisfaction predicts WFH success and ***air-quality-improving behavior*** *is associated with higher satisfaction."*

Reviewer #3

- I find your manuscript very interesting and timely. Having data supported information about the physical environment and its relationship to productivity is really crucial in the era of changing work environment.

We thank the reviewer for the kind words and appreciate the acknowledgement of the timely nature of our manuscript.

- However, you use correlational strategy, therefore, you should completely avoid using causal language in the manuscript.

We agree with the statement of the reviewer. We have carefully reviewed our manuscript and avoided any suggestion of causal language remaining. We highlight that we now mainly refer to our results as associations, for example:

"We find that higher home office satisfaction ***is associated with*** *WFH success and air-quality-improving behavior* ***is associated with*** *higher satisfaction."*

Note that only in the context of regression coefficients, do we refer to the associations as predictions. However, this term (like most regression coefficients) does of course not imply causality.

- Moreover, to make mediation results sound, you have to use cross-lagged design with at least two measurement point.

We appreciate and underwrite your concern about ensuring the robustness of our mediation results. A cross-lagged design with multiple measurement points is indeed considered the most robust approach for establishing temporal precedence and causality in mediation analysis (Cole & Maxwell, 2003; VanderWeele, 2015). However, we believe that our current design and analytical approach still provides sound and valid insights for the following reasons:

- *The relationship between air-quality, ventilation rate, and satisfaction and performance is well-established in the literature through both lab and field studies (for instance: Coley et al., 2007; Fang et al., 2004; Young et al., 2024). Our SEM is not designed to claim proof of the causal relationship between air-quality and performance. Instead, it focuses on the direct and indirect associations between ventilation, satisfaction, and performance.*

- *Second, precedents in the field suggest the value of mediation analysis with limited data when meeting literature-supported theoretical justification as mentioned above. Additionally, cross-sectional mediation analyses are perceived as a preliminary step towards understanding complex relationships (Achterberg et al., 2021; Baron & Kenny, 1986). We now highlight that our study provides crucial initial insights that pave the way for future longitudinal investigations.*
- *Finally, we are practically constrained by timing and high attrition in the online setup, limiting our ability to implement the multiple measurement points in the current study.*

We have amended the text to highlight the causal limitations through the following adjustments:

1) *In-text: "Following the main analysis, we implement a mediation analysis, using structural equation modelling.* ***This analysis of direct and indirect associations assesses*** *the impact of the physical environment on productivity, mediated by hardware and indoor environment satisfaction factors."*
2) *Footnote: "While our study employs a cross-sectional design, which limits the ability to establish causality compared to a cross-lagged design, extensive research from controlled experimental settings supports the relationship between ventilation and air quality and their effects on satisfaction, well-being, and performance.* ***Nevertheless, we remain cautious and consider our assessment an analysis of direct and indirect associations.*** *For more on this, see the Limitations section."*
3) *Limitation section: "Second, practical constraints limited our ability to implement a cross-lagged design with multiple measurement points which is considered the*

normative approach for establishing temporal precedence and causality in mediation analysis (60,61). Our study employed a cross-sectional design, which has precedent in the literature (62,63), and can still provide valuable insights. We justify our approach based on the model fit and both theoretical as well as literature support of the causal role of air quality improvement on satisfaction and performance (35,40,64). ***However, we acknowledge that this approach does not allow for the determination of causality with the same rigor as a longitudinal design. Future research should aim to utilize cross-lagged designs to further validate our findings and establish clearer causal relationships.***
