# Supplementary material for: Does working from home work? That depends on the home
Source: PLoS One. 2024 Aug 7;19(8):e0306475. doi: 10.1371/journal.pone.0306475 (PMC11305525; doi:10.1371/journal.pone.0306475)
Supplement: Attachment — Submitted filename: Response to Referees.docx [file pone.0306475.s007.docx]

**Response to Referees**

My main criticism concerns the way this manuscript is organised as well as its extensive length. In more detail, major concerns are as follows:

1. There is no justification of authors’ deviation from the usual structure of scientific papers (Introduction, Methods, Results, Discussion, Conclusions). In a rather strange way, authors mix up introductory parts with methods (p. 8ff of MS), and they mix up presentation of results with discussion (p. 23f.), while failing to present a separate Discussion section including strengths and limitations of own study.

*We have completely restructured the document as proposed by the reviewer. Specifically, we have moved the summary from the introduction to the discussion, restructured the methods and results section using level 1, 2 and 3 headers, and moved all interpretation of the results to the discussion section. Moreover, we have amended the limitation section.*

2. The manuscript is definitely too lengthy in its current version. Readers expect a more concise presentation of essential information. The whole part from p. 8 to p. 20 requires substantial cuts.

*We have made substantial cuts to the methods section and the descriptive part of the results section. The essential descriptions have been minimized. We did remove the descriptive table on home office versus the office comparison, but left some of the description of differences in as it relates to Table 2. Since this is the first paper to closely look at home office characteristics, we expect that this relatively simple comparison is unknown to the reader and will be of interest on its own.*

3. While authors put a lot of effort into methodological details, they do not reflect on limitations of their study, such as limited validity of self-reported data (e.g., common method variance between ‘predicting', ‘mediating’ and ‘outcome’ variables) or limited generalisation of findings (e.g., sample representativeness; COVID-19 specific effects). Authors use a biased language when mentioning ‘causal indicators’ (p. 25) or ‘burnout proneness’ (p. 22 and passim), suggesting availability of longitudinal data.

*We have amended the limitations in the discussion section to describe more elaborately and explicitly the limitations mentioned by the referee. Moreover, we acknowledge the referee’s comment that the preciseness of the language used is important, especially since our data limits the degree to which we can claim causality. As such, we replaced language as suggested by the referee with terms that do not suggest a casual effect (e.g. causal to predictive and propensity to tendency).*
